# Altered collecting duct adenylyl cyclase content in collecting duct endothelin-1 knockout mice

**DOI:** 10.1186/1471-2369-8-8

**Published:** 2007-05-23

**Authors:** Kevin A Strait, Peter K Stricklett, Donald E Kohan

**Affiliations:** 1 Division of Nephrology, University of Utah Health Sciences Center, 1900 East 30 North, Salt Lake City, UT 84132, USA

## Abstract

**Background:**

Endothelin-1 (ET-1) inhibition of vasopressin (AVP)-stimulated water reabsorption by the inner medullary collecting duct (IMCD) is associated with reduced cAMP accumulation. To determine the effect of ET-1 deficiency, AVP-stimulated cAMP responsiveness was assessed in IMCD from mice with collecting duct-specific deletion of ET-1 (CD ET-1 KO) and from control animals.

**Methods:**

Cyclic AMP production, adenylyl cyclase (AC) mRNA, and AC protein were measured in acutely isolated IMCD.

**Results:**

CD ET-1 KO IMCD had enhanced AVP-stimulated cAMP accumulation. Inhibition of calcium-stimulated AC using BAPTA did not prevent enhanced AVP responsiveness in CD ET-1 KO IMCD. Factors known to be modified by ET-1, including nitric oxide, cyclooxygenase metabolites, and superoxide did not affect the increased AVP responsiveness of CD ET-1 KO IMCD. Differential V2 receptor or G-protein activity was not involved since CD ET-1 KO IMCD had increased cAMP accumulation in response to forskolin and/or cholera toxin. CD ET-1 KO did not affect mRNA or protein levels of AC3, one of the major known collecting duct AC isoforms. However, the other known major collecting duct AC isoform (AC5/6) did have increased protein levels in CD ET-1 KO IMCD, although AC5 (weak signal) and 6 mRNA levels were unchanged.

**Conclusion:**

ET-1 deficiency increases IMCD AC5/6 content, an effect that may synergize with acute ET-1 inhibition of AVP-stimulated cAMP accumulation.

## Background

Endothelin-1 (ET-1) is likely to be an important regulator of water reabsorption by the collecting duct. The majority of studies done to date have utilized in vitro collecting duct preparations; in these experiments, exogenous ET-1 consistently inhibits vasopressin (AVP) action. ET-1 reduces AVP-enhanced water flux in acutely isolated rat cortical collecting tubules [1]. This effect is mediated, at least in part, by protein kinase C (PKC)-sensitive inhibition of adenylyl cyclase activity and is independent of dihydropyridine-type calcium channels and cyclooxygenase metabolites [2,3]. ET-1 also inhibits AVP-stimulated osmotic water permeability in in vitro perfused IMCD [4,5]. Similar to the cortical collecting tubule, the ET-1 effect is likely through reduction of AVP-stimulated cAMP accumulation [2,6]. Notably, ET-1 does not alter dibutyryl-cAMP-stimulated osmotic water permeability in the IMCD [5], indicating that the inhibitory effect of ET-1 in the IMCD is primarily due to reduction of adenylyl cyclase activity. Also like the cortical collecting duct, ET-1 inhibition of cAMP accumulation in the IMCD is PKC-dependent and is unrelated to cyclooxygenase or dihydropyridine-type calcium channel activity [2,4,6]. In addition, ET-1 acts through an inhibitory G protein since pertussis toxin blocks the effects of ET-1 on AVP-stimulated cAMP levels and water transport in IMCD [4,6]. Taken together, the above studies indicate that ET-1 is capable of acutely reducing collecting duct water reabsorption through inhibiting adenylyl cyclase activity.

ET-1 may also chronically regulate collecting duct water transport. Recent studies, using mice with collecting duct-specific knockout of ET-1 (CD ET-1 KO), observed reduced plasma AVP levels, enhanced AVP-stimulated cAMP accumulation in acutely isolated IMCD, and impaired ability to excrete an acute water load [7]. Thus, chronic deletion of ET-1 in the collecting duct resulted in increased sensitivity to the hydroosmotic and cAMP-stimulating effects of AVP. Whether this effect is due to reduced activity of signaling pathways implicated by acute in vitro studies to mediate ET-1 inhibition of AVP action, or if other mechanisms are involved, remains uncertain. Hence, the aim of the current study was to examine the mechanism(s) responsible for increased AVP sensitivity in collecting ducts of CD ET-1 KO mice. We report that chronic CD ET-1 deficiency results in increased AC5/6 protein content in IMCD.

## Methods

### Materials

Cholera toxin, pertussis toxin, BAPTA and tempol were obtained from Calbiochem (San Diego, CA). All primary antibodies directed against adenylyl cyclases were purchased from Santa Cruz Biotechnology (Santa Cruz, CA). Indomethacin, S-nitroso-N-acetylpenicillamine (SNP), nitroprusside, monomethylarginine (L-NMMA) and all other reagents were obtained from Sigma Chemical Co. (St. Louis) unless specifically stated otherwise.

### Transgenic mice

Mice with CD-specific disruption of the ET-1 gene were generated as previously described [7]. Mice containing the loxP-flanked (floxed) exon 2 of the ET-1 gene (obtained from Dr. M. Yanagisawa at University of Texas Southwestern) were mated with AQP2-Cre mice, the latter containing a transgene with 11 kb of the mouse AQP2 gene 5'-flanking region driving expression of Cre recombinase, an SV40 nuclear localization signal on the NH2 terminus of Cre, and an 11-amino acid Herpes Simplex virus epitope tag on the COOH terminus of Cre. Female AQP2-Cre mice were mated with male floxed ET-1 mice; female offspring heterozygous for both AQP2-Cre and floxed ET-1 were bred with males homozygous for floxed ET-1. Animals homozygous for floxed ET-1 and heterozygous for AQP2-Cre (CD ET-1 KO) were used in all studies. Sex-matched littermates that were homozygous for the floxed ET-1 gene, but without Cre, were used as controls in all studies.

### Genotyping

Tail DNA was PCR amplified for the AQP2-Cre transgene using oligonucleotide primers mAQP2 F (5'-CCTCTGCAGGAACTGGTGCTGG-3') and CreTag R (5'-GCGAACATCTTCAGGTTCTGCGG-3'), which amplify the 671-bp junction between the mouse AQP2 promoter and the Cre gene. The ET-1 gene was amplified using three primer sets. Primers ET-1CF (5'-GCTGCCCAAAGATTCTGAATTC-3') and ET-1BR (5'-GATGATGTCCAGGTGGCAGAAG-3') amplify 800 bp of the endogenous ET-1 allele. The primers ET-1AF (5'-CCCAAAGATTCTGAATTGATAACTTCG-3') and ET-1BR (5'-GATGATGTCCAGGTGGCAGAAG-3') amplify the same region, but the forward primer overlaps loxP and only recognizes the floxed ET-1 allele. Primers ET-1AF and ET-1DR (5'-AAC CTC CCA GTC CAT ACG GTA C-3') amplify the region spanning the loxP sites; the product of the non-recombined allele is 2 kb, while the recombined product is 300 bp.

### Acutely isolated IMCD preparations

Primary isolates of IMCD cells were obtained using a modification of a previously described procedure [6]. Mice were sacrificed by cervical dislocation, the kidneys removed, and the inner medulla minced and incubated in 0.1% collagenase (Type I; Worthington, Freehold, NJ) containing 0.01% DNase (type I) in Hanks' balanced salts solution (HBSS) supplemented with 15 mM HEPES (pH 7.4). Tissue digestion was carried out at 37°C in a shaking water bath. When a suspension containing predominantly single cells and individual tubules was obtained (approx. 45 min), the digest was filtered through a 74-μm-mesh screen to remove any residual tissue. The tubule suspension was centrifuged at 1500 rpm for 5 min, and the cell pellet resuspended in 10% bovine serum albumin in HBSS, followed by an additional 2 centrifuge/washes with HBSS. The final pellet containing primarily tubules was resuspended in HBSS/HEPES. This procedure has previously been shown to yield predominantly collecting ducts [6]. All studies were done comparing control to CD ET-1 KO IMCD on the same day and under the same isolation conditions.

### Cell culture

Freshly isolated IMCD were grown in primary culture as previously described [8]. The cell suspension was plated in 24 well plates in Renal Epithelial Cell Growth Media (Cambrex, Walkersville, MD) containing epidermal growth factor, insulin, hydrocortisone, gentamicin, amphotericin, 0.5% fetal bovine serum, epinephrine, triiodothyronine, and transferrin (concentrations are proprietary). Once cells reached confluence, the media was changed to DMEM:F12 alone for 16–24 hr prior to study.

### cAMP studies

Freshly isolated or cultured IMCD from floxed or CD ET-1 KO animals were pre-incubated in HBSS/HEPES buffer (pH 7.4) containing 1 mM 3-isobutyl-l-methylxanthine (IBMX) for 30 min at 37°C, followed by addition of 100 pM AVP, 1 nM AVP or 1 μM forskolin for 10 min. In studies involving additional treatments (inhibitors, etc), theses agents were added to the incubation at the same time as the IBMX. At the end of the incubations, cellular cAMP and total protein were determined. cAMP was extracted from cells by addition of 70% ethanol overnight at -20°C. The sample was then centrifuged and the ethanol removed from the cell pellet. The ethanol was evaporated, the residual material resuspended in assay buffer, and cAMP assayed using a commercial enzyme immunoassay kit (Assay Design, Ann Arbor, MI). The cell pellet (precipitated in ethanol) was re-dissolved in 0.1 N NaOH for determination of total cellular protein by the Bradford assay.

### Western analysis of adenylyl cyclases

IMCD were prepared as above, solubilized in Laemmli buffer containing 0.5% lithium dodecyl sulfate, and sonicated to reduce viscosity. Samples were run on a denaturing NUPAGE 4–12% Bis-Tris mini-gel using a MOPS buffer system (Invitrogen, Carlsbad, CA). Proteins were transferred to PVDF plus nylon membrane by electroelution and visualized using the Adance ECL system (GE Healthcare Piscataway, NJ). Protein loading was normalized to β-actin. Densitometry was done using a BioRad gel documentation system (Hercules, CA).

Anti-adenylyl cyclase antibodies used for immunoblotting were incubated with membranes in blocking buffer (5% milk-TBST) overnight at 4°C at concentrations from 1:25,000–1:100,000. Secondary donkey anti-rabbit horseradish peroxidase-conjugated antibody (GE Healthcare, Piscataway, NJ) was incubated at room temperature in blocking buffer. After visualization, blots were stripped and incubated with anti-β-actin antibody (Abcam, Cambridge, MA) at 1:10,000 in blocking buffer, then exposed to secondary antibody as above.

### RT-PCR of adenylyl cyclase mRNA

Total RNA was prepared from isolated IMCD using acid phenol, and potentially contaminating genomic DNA removed by incubation with RNase-free DNase I (Promega, Madison, WI). Reverse transcription was performed on 3 μg total RNA using oligo (dT)_12–18 _and Superscript II according to the manufacturer's protocol (Invitrogen Carlsbad, CA). PCR primers were designed from mouse cDNA sequences to span an intron in order to distinguish genomic contamination. PCR amplification was performed using Taq polymerase (Invitrogen). All products were sequenced to insure fidelity of amplification. Primers used were: AC3 F5'-GCAGCACCTGGCTGACCTGGCTGA-3' and R5'-TGGGGCAGTGTAACAGAGGAGCCA-3' which yields a 383 bp product; AC5 – F5'-CAATACAGTGAATGTGGCCAGCCG-3' and R5'-TTCCCTTACAGGGCATTGTCTCTG-3' product which yields a 363 bp product; AC6 -F5'-GATTCCGGCAGTTGGAGAAGATCA-3' and R5'-TCCCACTCAATGCCCACCTTGGTC-3' product which yields a 494 bp product; and GAPDH – F5'-CCTTCATTGACCTCAACTACATGG-3 and R5'-GCAGTGATGGCATGGACTGTGGT-3' which yields a 442 bp product. Gels were stained with ethidium bromide and mRNA content determined by densitometry. All results were normalized to GAPDH.

### Statistics

Comparisons of cAMP accumulation or adenylyl cyclase mRNA or protein between CD ET-1 KO and floxed mice for a given condition, or between AVP and AVP + inhibitor in floxed mice, were made by the unpaired Student's t-test. p < 0.05 was taken as significant. Data are expressed as mean ± SEM.

## Results

Acutely isolated mouse IMCD accumulate detectable cAMP only in the presence of phosphodiesterase inhibition; consequently, all studies were done in the presence of IBMX. No cAMP was detectable in the absence of agonists or in the presence of vehicle for drugs alone. AVP was added at a concentration of 1 nM for 10 minutes since this regimen has previously been determined to cause half-maximal stimulation of cAMP accumulation in IMCD from floxed and CD ET-1 KO animals [7]. Forskolin was used at a maximal cAMP stimulatory concentration of 1 *μ*M [7]. Tubules were exposed to IMBX and various inhibitors/reagents for 30 minutes before addition of AVP or forskolin -incubation of acutely isolated IMCD for longer periods led to reduced AVP or forskolin sensitivity, presumably due to decreased viability of acutely isolated cells in suspension. Thus, while failure of inhibitors to exert an observable effect on AVP or forskolin responsiveness might be attributed to the length of exposure to the cells, these were the only workable conditions. Notably, primary cultures of mouse IMCD have markedly decreased AVP-stimulated cAMP accumulation, and the difference in AVP, as well as forskolin, responsiveness between floxed and CD ET-1 KO mice was lost (1 *μ*M forskolin: CD ET-1 KO 6.91 ± 1.00 vs. control 6.38 ± 1.8 nmol cAMP/*μ*g total cell protein; 100 nM AVP: CD ET-1 KO 0.94 ± .35 vs. control 1.71 ± 0.81 nmol cAMP/*μ*g total cell protein). Hence, only acutely isolated preparations were studied. It should also be noted that 100 pM AVP also caused enhanced CD ET-1 KO cAMP accumulation (157 ± 9% of control values, n = 15, p < 0.001). In addition, results using 1 nM AVP are described below, however similar results were obtained using 100 pM AVP (data not shown).

**Figure 1:**
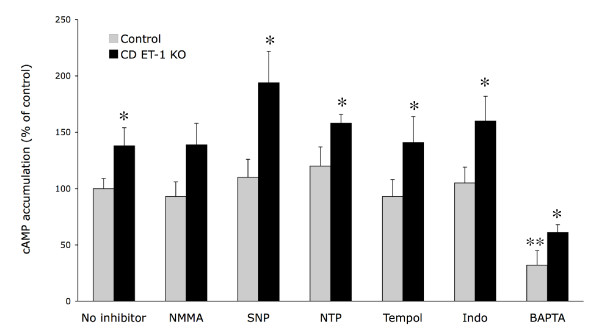
Effect of 100 *μ*M NMMA, 1 mM SNAP (SNP), 1 mM nitroprusside (NTP), 5 mM tempol, 10 *μ*M indomethacin (Indo), or 1 *μ*M BAPTA on AVP (1 nM) – stimulated cAMP accumulation in acutely isolated IMCD from CD ET-1 KO and floxed (control) mice. All data are expressed as percent of cAMP accumulation with AVP alone in control IMCD. Control IMCD AVP-stimulated cAMP accumulation was 141 ± 9 fmol cAMP/*μ*g total cell protein (n = 18). *p < 0.05 compared to floxed controls treated with the same condition, **p < 0.01 vs. control + no inhibitor. N = 6–18 each data point.

IMCD from CD ET-1 KO, as compared to floxed, mice had approximately 40% greater cAMP accumulation in response to AVP, as previously reported [7]. Inhibition of NOS with NMMA, or addition of SNP or NTP, at concentrations previously shown to inhibit IMCD NO production or to increase IMCD cGMP content, respectively [9], did not alter AVP-stimulated cAMP accumulation in CD ET-1 KO IMCD or floxed controls (Figure 1). Tempol, at a concentration shown to prevent superoxide production in collecting duct (5 mM) [10], did not affect AVP responsiveness in IMCD from either group of animals (Figure 1). Indomethacin, at a concentration shown to inhibit PGE2 production in IMCD (10 *μ*M) [6], did not alter AVP-augmented cAMP content in CD ET-1 KO or floxed IMCD (Figure 1). Thus, inhibition of NO, superoxide and or prostaglandin formation did not affect AVP-stimulated cAMP accumulation.

**Figure 2:**
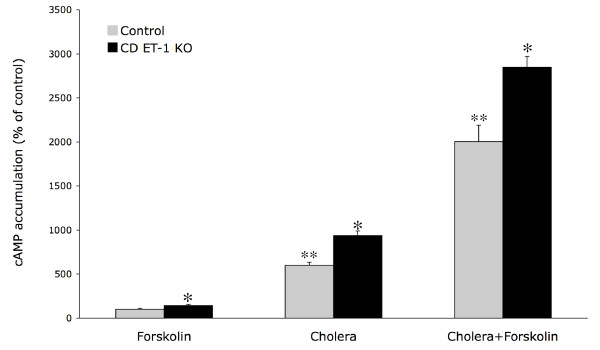
Effect of 1 *μ*M forskolin, 1 *μ*g/ml cholera toxin, or both agents together on cAMP accumulation in acutely isolated IMCD from CD ET-1 KO and floxed (control) mice. All data are expressed as percent of cAMP accumulation with forskolin alone in control IMCD. Control IMCD forskolin-stimulated cAMP accumulation was 497 ± 32 fmol cAMP/*μ*g total cell protein (n = 15). *p < 0.05 compared to floxed controls treated with the same condition; **p < 0.005 vs. forskolin alone in control IMCD. N = 5–10 each data point.

Augmented cAMP levels persisted in IMCD from CD ET-1 KO IMCD treated with either pertussis or cholera toxin (172 ± 18% and 142 ± 6%, respectively, of similarly treated floxed IMCD) (Figure 2), indicating that the enhanced AVP responsiveness in CD ET-1 KO IMCD is not due to alterations in the V2 receptor or G proteins. Further evidence of this was the finding that 1 *μ*M forskolin increased cAMP accumulation in CD ET-1 KO IMCD 165 ± 11% (n = 19, p < 0.001) as compared to control IMCD (Figure 2), in agreement with previous findings [7]. Even combining forskolin with cholera toxin, which caused a 40-fold increase in cAMP levels over that seen with AVP alone, resulted in enhanced CD ET-1 KO cAMP levels (142 ± 10% of controls, n = 8, p < 0.05) (Figure 2).

**Figure 3:**
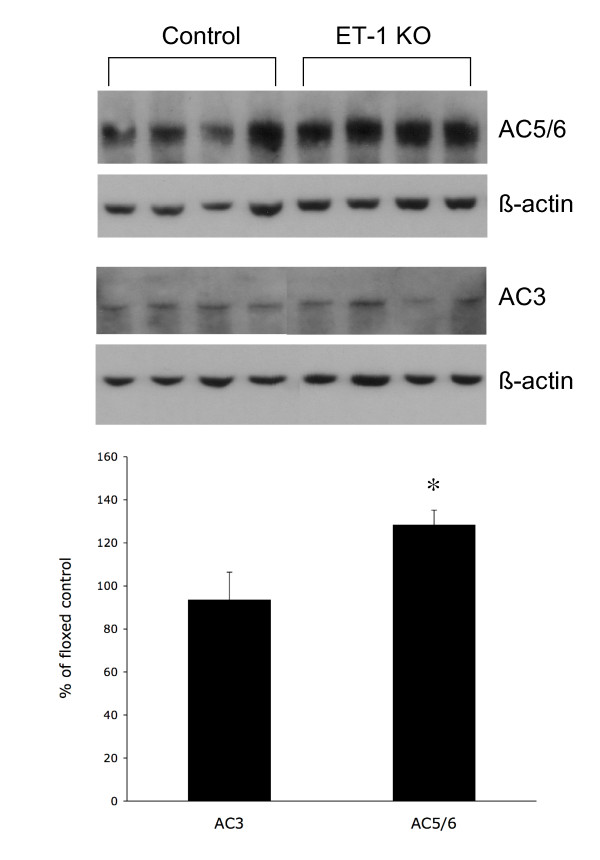
Western analysis of adenylyl cyclases 3 (AC3) and 5/6(AC5/6) in floxed control and CD ET-1 KO IMCD. All results were normalized to β-actin. Upper panel shows representative blot (n = 8 total). Bottom panel shows densitometry analysis. *p < 0.05 CD ET-1 KO IMCD AC protein content as compared to that observed in floxed controls.

**Figure 4:**
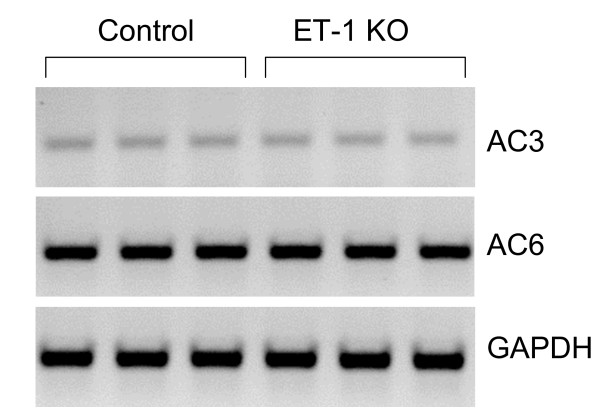
RT-PCR analysis of adenylyl cyclases 3 (AC3) and 6 (AC6) in floxed control and CD ET-1 KO IMCD. All results were normalized to GAPDH. A representative blot is shown (n = 5 total).

Chelation of intracellular calcium with BAPTA reduced AVP-stimulated cAMP accumulation, however it failed to prevent enhanced AVP responsiveness in CD ET-1 KO mice (Figure 1). Of the three known calcium-stimulated adenylyl cyclases (1, 3 and 8), IMCD express primarily AC3 mRNA [11]. There was no difference in AC3 protein between CD ET-1 KO and floxed IMCD (Figure 3); similarly, the total AC3 mRNA levels were not different between IMCD from the two groups (Figure 4). Thus, calcium – dependent adenylyl cyclase activity, which most likely reflects AC3 activity, does not account for enhanced AVP responsiveness in CD ET-1 KO IMCD.

The failure to see an effect of acute inhibitor exposure on the enhanced AVP responsiveness of CD ET-1 KO IMCD suggested that the total amount of one or more adenylyl cyclases may be increased in these cells. Using Western analysis, we were only able to detect AC3 (as stated above) and AC5/6 (the only antibody available to us can not distinguish between AC5 and AC6) protein. Notably, AC5/6 protein was significantly increased in IMCD from CD ET-1 KO mice (130% of floxed controls) (Figure 3). PCR of IMCD revealed a faint signal for AC5 (data not shown) and a relatively strong signal for AC6 (Figure 4). However, CD ET-1 KO mice did not have significantly different AC6 mRNA content (Figure 4).

## Discussion

Collecting duct-specific deficiency of ET-1 is associated with reduced plasma AVP levels and impaired ability to excrete an acute water load [7]. This effect is due, at least in part, to enhanced AVP-stimulated cAMP accumulation in ET-1-deficient IMCD. In the current study, we found that acute blockade of nitric oxide synthase, superoxide formation, cyclooxygenase, or intracellular calcium failed to prevent the exaggerated AVP responsiveness in CD ET-1 KO mice. These observations suggested that there might be an absolute increase in adenylyl cyclase protein content. Indeed, CD ET-1 KO caused an increase in adenylyl cyclase 5/6 protein content, and this increase was of a magnitude comparable to that seen for increased AVP-stimulated cAMP levels (CD ET-1 KO IMCD have approximately a 40% greater AVP-stimulated cAMP content and 30% greater AC5/6 levels as compared to floxed controls). Thus, chronic deficiency of ET-1 in the collecting duct increases adenylyl cyclase protein; taken together with the known acute inhibitory effect of ET-1 on AVP-stimulated cAMP accumulation, these results suggest that collecting duct-derived ET-1 may exert a diuretic effect through both acute modulation of adenylyl cyclase activity and chronic down-regulation of adenylyl cyclase protein content.

The mechanism(s) by which ET-1 deficiency leads to elevated adenylyl cyclase levels are unknown. We detected a faint signal for AC5 mRNA and a much stronger signal for AC6. Such findings are in agreement with two previous studies wherein AC6 mRNA was strongly present in rat IMCD, but AC5 mRNA was undetectable [12,13]. Another study found that rat collecting duct principal cells expressed AC6, but not AC5 mRNA, while intercalated cells expressed message for both isoforms [14]. Thus, while our Western analysis is not conclusive, taken together with the apparently very low AC5 mRNA signal in mouse IMCD, and essentially no AC5 mRNA in rat IMCD, the data suggest that AC6 is the predominant isoform undergoing ET-1 regulation. Interestingly, no change in AC6 mRNA was identified in CD ET-1 KO mice. This could represent translational regulation of AC6 mRNA, increased trafficking of AC6 to the membrane, or reduced metabolism of AC6 protein. Another possibility is that the changes in AC6 mRNA that would account for a 30% increase in AC6 protein would be very difficult to detect. It is worth noting, however, that there is precedent for changes in AC6 content in IMCD – Hoffert and colleagues have reported a 5-fold decrease in AC6 protein abundance in inner medulla from Brattleboro rats given dDAVP as compared to saline alone [11]. Finally, the signaling pathways by which ET-1 modulates adenylyl cyclase protein content are unknown; determine of these regulatory systems is in need of further investigation.

In this study, forskolin stimulated cAMP accumulation in IMCD, however CD ET-1 KO IMCD had greater sensitivity to forskolin than did control IMCD, in agreement with previous observations [7]. Cholera toxin augmentation of cAMP levels was also greater in IMCD from CD ET-1 KO mice. When cAMP accumulation was maximally and massively stimulated by combining cholera toxin and forskolin, IMCD from CD ET-1 KO still exhibited greater AC responsiveness. Notably, the percent of enhanced responsiveness in CD ET-1 KO IMCD was similar in AVP- and forskolin/cholera toxin-treated cells. Taken together, these data strongly suggest that neither the V2 receptor nor G-proteins can account for the increased cAMP levels in CD ET-1 KO mice, supporting a primary change in AC activity per se.

Finally, the question must be raised as to whether ET-1 regulates mouse collecting duct water transport in a manner similar to that described in other species. Relatively few studies have examined ET-1 actions in mouse collecting duct, perhaps due to technical difficulties. We did observe in our study on water transport in CD ET-1 KO mice [7] that absence of CD ET-1 caused a reduced ability to excrete an acute water load associated with increased urine osmolality. Notably, and as in other species, ET-1 increased intracellular calcium concentration in mouse Ml CCD cells by stimulating both release from intracellular stores as well as nifedipine-sensitive calcium entry [15]. Similarly, Naruse and colleagues found that ET-1 increased intracellular calcium concentration in superfused mouse CCD, OMCD, and IMCD. Taken together with the findings in the current study, these investigations strongly suggest that mouse collecting function, including water transport, is regulated by ET-1.

## Conclusion

Collecting duct-specific deletion of ET-1 is associated with an increase in most likely AC6 protein abundance. This increase in AC6 is associated with a comparable degree of increase in collecting duct AVP-stimulated cAMP accumulation. Thus, ET-1 may be capable of both acute and chronic inhibition of collecting duct AVP responsiveness. Such a system may be important in the regulation of collecting duct water reabsorption, particularly under water diuresis conditions wherein elevated collecting duct ET-1 may facilitate net renal water excretion.

## Competing interests

The author(s) declare that they have no competing interests.

## Authors' contributions

K.A.S. performed the cAMP studies. P.K.S. performed the RT-PCR and Western analyses. D.E.K. designed the study, analyzed and interpreted the data, and wrote the manuscript.

## Pre-publication history

The pre-publication history for this paper can be accessed here:


